# Incidental finding in a newborn with respiratory distress

**DOI:** 10.1590/S1679-45082017AI4001

**Published:** 2017

**Authors:** Natália Noronha, Pedro Pires Epifânio, Patrícia Vaz Silva, António Pires, António Marinho, Eduardo Castela

**Affiliations:** 1 Hospital Pediátrico, Centro Hospitalar e Universitário de Coimbra, Coimbra, Portugal.

A full-term 1-day-old male newborn was admitted to the neonatal intensive care unit due to respiratory distress and cyanosis. The sepsis screen was negative and he was diagnosed as transient tachypnea of the newborn. He required supplemental oxygen for the following days and the sepsis screen was repeated and was negative. He remained in respiratory distress, particularly during feeding, and was referred to a pediatric cardiologist.

**Figure 1:**
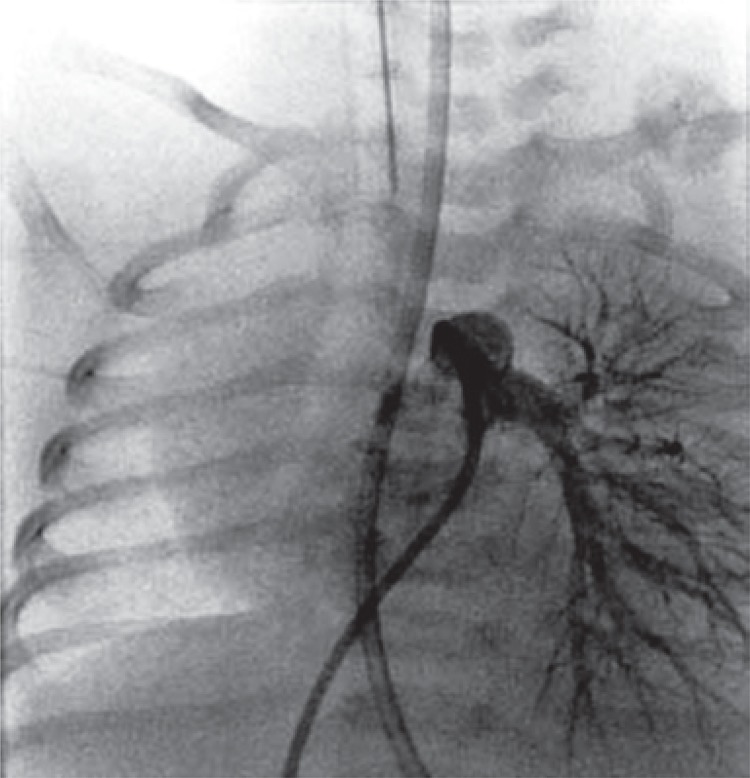
Pulmonary angiography showing absent right pulmonary artery and patent left pulmonary artery branches

**Figure 2:**
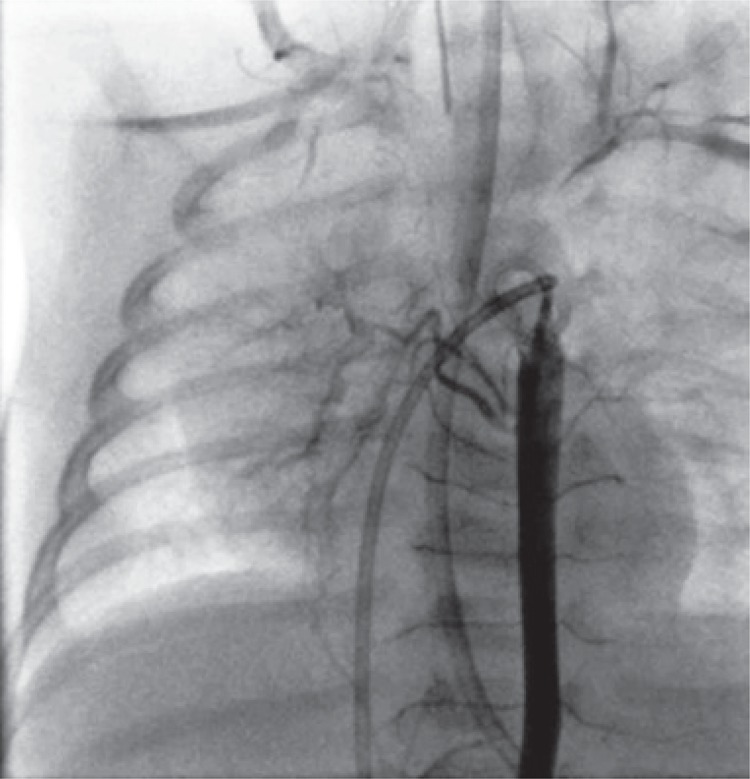
Aortic angiography showing exuberant collateral circulation of the right lung

On transthoracic echocardiography, the heart appeared to be structurally and functionally normal. However the right pulmonary artery was not clearly evident. A computerized tomography angiography was performed to clarify this finding, but once again it was inconclusive. Nevertheless, hemitruncus and patent ductus arteriosus were ruled out. Subsequently he was catheterized and the pulmonary angiography showed agenesis of the right pulmonary artery (Figure 1), ruling out occult pulmonary branch. The right lung was perfused by the bronchial arterial network (Figure 2) and no other anomalies were found.

He was gradually weaned off oxygen and was discharged home after 7 days. He is currently asymptomatic.

Unilateral absence of the pulmonary artery is a rare congenital lesion with an estimated prevalence of 1/200,000.^(1)^ It is generally associated with other cardiovascular anomalies, although isolated form may also occur.^(2)^ The latter is rarely diagnosed during the neonatal period. The signs may be subtle and easily missed and a high index of suspicion is required to diagnose this entity. In the absence of cardiopulmonary dysfunction, no treatment is required. However, regular follow-up is advised.^(3)^
